# Increased Risk of Acute Pancreatitis in Patients with Chronic Hemodialysis: A 4-Year Follow-Up Study

**DOI:** 10.1371/journal.pone.0071801

**Published:** 2013-08-20

**Authors:** Sheng-Wen Hou, Yi-Kung Lee, Chen-Yang Hsu, Ching-Chih Lee, Yung-Cheng Su

**Affiliations:** 1 Emergency Department, Shin-Kong Wu Ho-Su Memorial Hospital, Taipei, Taiwan; 2 Emergency Department, Buddhist Tzu Chi Dalin General Hospital, Chiayi, Taiwan; 3 School of Medicine, Tzu Zhi University, Hualien, Taiwan; 4 Department of Public Heath, National Taiwan University, Taipei, Taiwan; 5 Community Medicine Research Center and Institute of Public Health, National Yang-Ming University, Taipei, Taiwan; 6 Department of Otolaryngology, Buddhist Dalin Tzu Chi General Hospital, Chiayi, Taiwan; 7 Cancer Center, Buddhist Dalin Tzu Chi General Hospital, Chiayi, Taiwan; Klinikum rechts der Isar der TU München, Germany

## Abstract

**Background:**

The risk of acute pancreatitis in patients on long-term peritoneal dialysis is higher as compared to the general population. However, the relationship between long-term hemodialysis and acute pancreatitis has never been established.

**Objectives:**

We investigated the incidence of acute pancreatitis among patients on long-term hemodialysis in Taiwan to evaluate if there is a higher risk of acute pancreatitis in comparison to the general population.

**Methods:**

We utilized a National Health Insurance (NHI) claims data sample containing one million beneficiaries. We followed all adult beneficiaries from January 1, 2007 until December 31, 2010 to see if they had been hospitalized for acute pancreatitis during this period. We further identified patients on chronic hemodialysis and compared their risk of acute pancreatitis with the general population.

**Results:**

This study included 2603 patients with long-term hemodialysis and 773,140 patients without hemodialysis. After controlling for age, gender, Charlson Comorbidity Index Score, geographic region, socioeconomic status and urbanization level, the adjusted hazard ratio was 3.44 (95% Confidence interval, 2.5–4.7).

**Conclusions:**

The risk of acute pancreatitis in patients on long-term hemodialysis is significantly higher in comparison to the general population.

## Introduction

Pancreatic abnormalities are commonly reported in chronic hemodialysis (HD) at postmortem autopsies [1], [2]. In one study, histological evidence of chronic pancreatitis was found in over half of these patients [1]. The high prevalence of pancreatic pathology after long-term dialysis hypothesized to some that dialysis might be also a risk factor of acute pancreatitis (AP). However, few studies have been conducted among patients with end-stage renal disease (ESRD). While patients receiving peritoneal dialysis (PD) were found to have a higher risk of obtaining AP in comparison to the general population [3]–[6], studies evaluating the incidence of AP in HD patients have been inconclusive.

In 2000, Bruno et al. reported the AP risks were not different between HD patients and the general population, but the case numbers of enrolled patients in the study were small; only one of 269 HD patients had an attack during the study period [3]. In 2008, another study in Germany suggested patients who underwent HD may have a higher incidence rate of AP; however, it was subject to recall bias inherited from the questionnaire design [6].

Our study investigated the correlation between long-term HD and AP by utilizing a large administration database to overcome the above-mentioned obstacles. Not only was recall bias avoided, but there were also enough observations to adjust various baseline differences and important confounding factors. Adjusted hazard ratios (HRs) and cumulative hazard ratios were used to present the risk of the first pancreatitis attack over a four-year period in comparing HD patients and control groups. In-hospital mortalities were also assessed in each group. Results of this study provide clinicians with further insights into this frequently encountered disease.

## Methods

### Ethics statement

This study was initiated after approval from the Institutional Review Board of Buddhist Dalin Tzu Chi General Hospital, Taiwan. Since all personal identification was stripped from the secondary files before analysis, the review board waived the requirement for written informed consent from the involved patients.

### Database

The National Health Insurance (NHI) program was implemented in Taiwan in 1995 and provides compulsory universal health insurance. It enrolls up to 99% of the Taiwanese population and contracts with 97% of all medical providers [7], [8]. The database contains comprehensive information on all insured subjects, including sex, date of birth, residential or work area, dates of clinical visits, the International Classification of Diseases (Ninth Revision) Clinical Modification (ICD-9-CM) diagnostic codes, details of prescribed medications, expenditure amounts and outcome at hospital discharge (i.e. recovered, died, or transferred out). A random sample comprised of 1,000,000 people based on the 2005 reimbursement data was established for public access; the group did not significantly differ statistically from the larger cohort in age, gender or health care costs, according to the Taiwan National Health Research Institute [8]–[10]. The sampled group was used as our study cohort.

### Study Population

**Figure 1:**
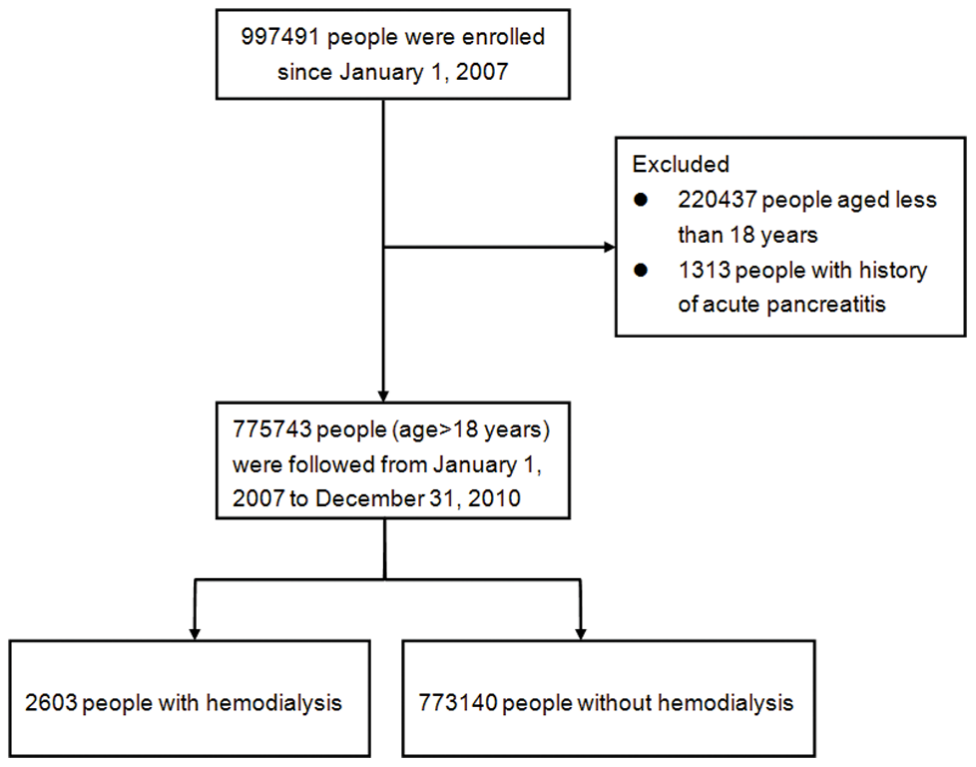
Flow diagram of the population-based study.

The sampled population was followed from January 1, 2005 to December 31, 2010 (a total of six years). First, we identified people as our study cohort who were still alive in 2007 and were older than 18 years of age. Chronic HD was defined when the total dialysis period was longer than six weeks before January 1, 2007 [3]. The AP was defined by ICD-9-CM code 577.0 in any position of the diagnoses. In order to maximize case ascertainment, only patients hospitalized for AP were included [11]. We excluded those with any cause of AP diagnosed before January 1, 2007. After exclusion of our cohort cases, we identified 2603 people with chronic HD and 773,140 without regular HD based on payment records. Each person was tracked for a four-year period from January 1, 2007 till December 31, 2010 in order to identify if he or she was diagnosed with AP. These patients were subsequently linked to the administrative data for the period 2007–2010 in order to calculate disease-free survival time. Cases were censored for patients who either drew back guarantees from the NHI Program or were still robust at end of the follow-up period(Figure 1).

### Covariates

In order to better understand the risk of chronic HD on AP, this study included several covariates. These included patient demographics such as age, sex, urbanization level (i.e. urban, suburban, and rural areas), geographic region of residence (i.e. Northern, Central, Southern and Eastern Taiwan) and socio-economic status (SES). This study used the income-related insurance payment amounts as a proxy measure of individual SES at follow-up. People were classified into three groups: (1) low SES: payment lower than US$571 per month (New Taiwan Dollars [NT$] 20000); (2) moderate SES: payment between US$571–1141 per month (NT$ 20,000–40,000); and (3) high SES: US$1142 or more payment per month (NT$40,001) or more [12]. Second, the prevalence of selected comorbid conditions (i.e. biliary tract disease, history of alcohol intoxication, diabetes and hyperlipidemia) and Charlson comorbidity index (CCI) were identified according to discharge diagnosis either during outpatient clinics visits or hospitalizations before January 1, 2007. The CCI is a scoring system that includes weighing factors on important concomitant diseases; it has been validated for use with ICD-9-CM coded administrative databases [13], [14]. Because HD is a variable of primary interest in this study, chronic renal failure was not counted in the CCI. We also tracked the outcome of each patient admitted to find out if he or she was discharged alive or post-mortem.

### Statistical analysis

The SAS statistical package, version 9.2 (SAS Institute, Inc., Cary, NC, USA), and STATA version 11.2 (StataCorp, College Station, TX, USA) were both used for data analysis. Pearson's chi-square test was used for categorical variables. The Nelson-Aalen cumulative hazard estimates for AP were plotted to show different trends between chronic HD patients and those without HD. A Cox proportional hazard regression model was subsequently used to calculate the AP hazard ratio for people with chronic HD after adjustments for age, gender, urbanization level, geographic region of residence, SES, diabetes, hyperlipidemia, history of alcohol intoxication, biliary tract disease, and CCI.

In order to further assess the robustness of our results, we also performed sensitivity analyses [15] to evaluate how large the effect of an unmeasured confounder would be for accounting for the results. A two-tailed P value of <0.05 was considered significant.

## Results

**Figure 2:**
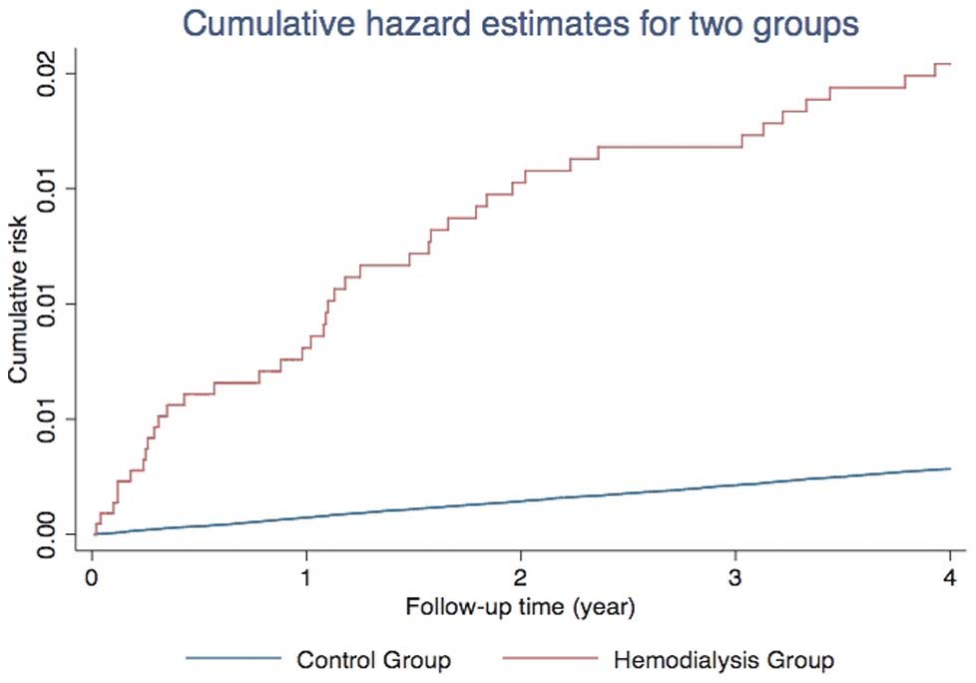
Nelson-Aalen cumulative risk curves.

The distribution of both demographic characteristics and selected morbidities is shown in Table 1. There were 2603 patients in the HD group and 773,140 in the control group. Total follow-up periods in the two groups were 7888 and 2,982,431 person-years, respectively. Patients with chronic HD were significantly older and also more likely to have diabetes, hyperlipidemia, biliary tract disease, higher CCI and lower socioeconomic status. At the end of follow-up, 2173 patients had been admitted for first-attack AP, with 41 patients in the chronic HD group and 2132 without chronic HD. The crude hazard ratio (HR) of AP between the two groups was 7.26 (95% Confidence interval [CI], 5.3–9.9), and the Nelson-Aalen plot showed a higher cumulative risk in the HD group. (Figure 2) The mortality rate in the HD group was also much higher in comparison to the control group. (7.3% versus 2.1%, p<0.001) For patients who had been hospitalized, the AP recurrence rates in the four years were similar. (22.0% in HD group versus 24.1% in the control group, p = 0.752).

**Table 1:** Baseline characteristics of the hemodialysis group and the control group.

Variables	Hemodialysis Group (n = 2603)		Control Group (n = 773140)		*P*-value
	No.	%	No.	%	
Gender					0.515
Male	1277	(49.1)	374359	(48.4)	
Patient age					<0.001
18–44 yrs	301	(11.6)	422196	(54.6)	
45–54 yrs	480	(18.4)	152331	(19.7)	
55–64 yrs	617	(23.7)	89306	(11.6)	
65–74 yrs	673	(25.9)	60084	(7.8)	
75 yrs and more	532	(20.4)	49223	(6.4)	
Charlson Comorbidity Index Score					<0.001
0	360	(13.8)	515154	(66.6)	
1	640	(24.6)	153852	(19.9)	
2	683	(26.2)	45481	(5.9)	
3–5	254	(9.8)	11318	(1.5)	
6–8	596	(22.9)	42515	(5.5)	
≥9	70	(2.7)	4820	(0.6)	
Geographic region					<0.001
Northern	1350	(51.9)	446820	(57.8)	
Central	464	(17.8)	138061	(17.9)	
Southern	717	(27.6)	171059	(22.1)	
Eastern	72	(2.7)	17200	(2.2)	
Diabetes	1046	(40.2)	60116	(7.8)	<0.001
Hyperlipidemia	586	(22.5)	75489	(9.8)	<0.001
History of alcohol intoxication	19	(0.7)	6102	(0.8)	0.733
Biliary tract disease	33	(1.3)	3967	(0.5)	<0.001
Socioeconomic status					<0.001
Low	1599	(61.4)	360011	(46.6)	
Moderate	873	(33.5)	298853	(38.7)	
High	131	(5.0)	114276	(14.8)	
Urbanization level					<0.001
Urban	725	(27.9)	241701	(31.3)	
Suburban	1132	(43.5)	330382	(42.7)	
Rural	746	(28.7)	201057	(26.0)	
First-attack acute pancreatitis	41	(1.6)	2132	(0.3)	<0.001
Mortality among pancreatitis	3	(7.3)	45	(2.1)	<0.001

Next, we performed the multivariate Cox regression model to evaluate the adjusted HRs of first-attack AP. Patients with chronic HD still had significantly high HR after controlling for age, sex, urbanization level, geographic region of residence, SES, biliary tract disease, history of alcohol intoxication, diabetes, hyperlipidemia and CCI. (3.44; 95% CI, 2.5–4.7) Other independent risk factors for AP included male gender, older age, higher CCI, diabetes, hyperlipidemia, biliary tract disease, history of alcohol intoxication, rural residency, and lower SES. All statistical results are summarized in Table 2.

**Table 2:** Adjusted Hazard Ratios (HR) of Admissions Because of Acute Pancreatitis.

Variables	Hazard ratio	95% confidence interval	P-value
Hemodialysis	3.44	2.52–4.70	<0.001
Male	1.85	1.70–2.03	<0.001
Patient age			
18–44 yrs	1	–	–
45–54 yrs	1.31	1.16–1.49	<0.001
55–64 yrs	1.40	1.21–1.60	<0.001
65–74 yrs	1.89	1.64–2.18	<0.001
75 yrs and more	2.72	2.36–3.13	<0.001
Charlson Comorbidity Index Score			
0	1	–	-
1	1.79	1.59–2.00	<0.001
2	2.30	1.98–2.66	<0.001
3–5	2.37	1.87–3.00	
6–8	2.08	1.78–2.43	
≥9	2.41	1.68–3.46	
Geographic region			<0.001
Northern	1	–	–
Central	1.18	1.05–1.33	0.005
Southern	1.09	0.97–1.22	0.137
Eastern	1.50	1.20–1.87	<0.001
Diabetes	1.37	1.22–1.54	<0.001
Hyperlipidemia	1.36	1.22–1.52	<0.001
History of alcohol intoxication	5.86	4.92–6.99	<0.001
Biliary tract disease	2.36	1.75–3.19	<0.001
Socioeconomic status			
Low	1	–	–
Moderate	1.03	0.94–1.13	0.544
High	0.65	0.55–0.77	<0.001
Urbanization level			
Urban	1	–	–
Suburban	1.17	1.04–1.31	0.009
Rural	1.48	1.30–1.68	<0.001

**Figure 3:**
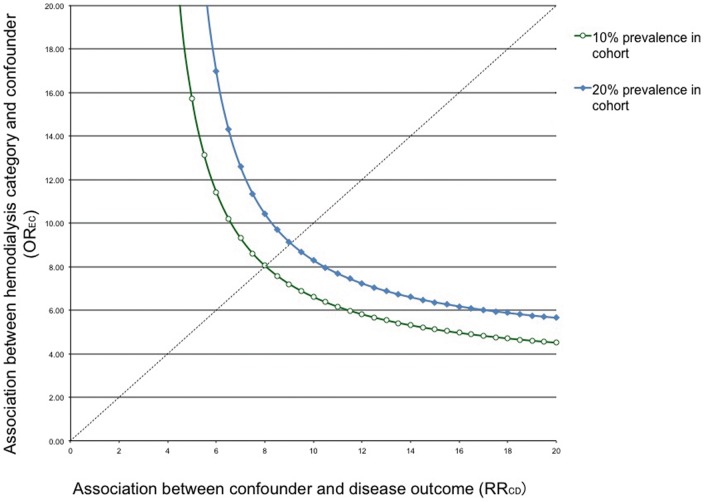
Sensitivity analyses of an unmeasured confounding.

Sensitivity analyses showed that an unmeasured confounder present in 10% of the study population would be required in order to elevate the AP risk by a factor of 8 and would also have to have a prevalence among patients with chronic HD that would be eight times that among general population to explain a lower 95% confidence limit HR of 2.5. (Figure 3).

## Discussion

In this study, we presented the largest cohort study to date to prove uremic patients who underwent HD had a higher risk of AP in comparison to the general population. A major textbook lists renal failure as an uncommon cause of AP [16], possibly owing to the use of drugs, uremia, and secondary hyperparathyroidism accompanied by hypercalcemia [3]. However, ESRD patients undergoing dialysis have different epidemiologic risks than renal failure patients; before our study there was conflicting evidences for a higher AP risk among HD patients.

Several studies confirmed the higher incidence of AP in patients receiving PD, either in comparison to HD patients or in the general populations [3], [4]. For patients receiving PD, it has been hypothesized that both elevated abdominal pressure during PD and a non-physiological composition solution may contribute to premature proteolytic enzymes activation, leading to an AP attack [3]. On the other hand, patients who underwent HD were not subjected to either of these two effects. Since both uremia and hypercalcemia improved after HD, it is worth knowing if these patients still had higher risks of AP.

In patients undergoing HD, the gastrointestinal hormones such as cholecystokinin, serum gastric inhibitory polypeptide, and glucagon are persistently increased, consequently causing over-secretion of pancreatic enzymes and possibly leading to impaired pancreatic function and pancreatic abnormalities [17]–[19]. We hypothesized the effects of HD on pancreas might be both chronic and acute. Chronic pancreatic abnormalities in HD patients have been well established based on postmortem autopsies, [1], [2] and our study also found out that HD was highly associated with acute flare up of pancreatitis.

After controlling for the numerous covariates, our study revealed a strong HR (3.44; 95% CI, 2.5–4.7) in HD group, which was statistically and clinically significant even at the lower end of CI. Unmeasured confounders, if they ever existed, would have difficulty in altering results at this scale. The database itself was validated and representative in relation to the original population data. Our study outcomes were also consistent with previous study findings, using the same database which showed that age, alcohol use, biliary disease and diabetes mellitus increases the risk of AP [11].

Another advantage of this study was its differentiation from previous studies which compared AP incidences with the general population [6]. In contrast, we directly compared HD patients with the general population simultaneously by survival analysis. Although the temporal relationship is sometimes difficult to assess in retrospective cohort studies, this study defined the HD group and general population group using the 2007 data. Subsequently, it tracked down patient results over four years through December 31, 2010. Thus, the exposure (i.e. HD) definitely occurred before AP episodes.

Some may argue that during the four-year follow-up, it was possible for some people originally belonging to the general population group may require HD later. The crossover extent in the exposure category was not assessed in this study, because under the intention to treat analysis principle, the crossover could only show a bias toward a null result, and the estimation of HR was more conservative than the actual number.

### Limitations

Several limitations were associated with the study. First, our findings were derived from administrative data. Cases were collected using ICD-9-CM diagnosis codes, which is good for insurance reimbursement instead of being the substitute for precise operative definition. Therefore, the validity of the diagnosis (i.e. sensitivity, specificity and accuracy) was not fully assessed. It is well known that amylase, the most frequently used laboratory test for AP, was metabolized more slowly in uremic patients, resulting in hyperamylasemia. Thus, it is sometimes difficult to make the precise AP diagnosis among HD patients; clinicians solely relying on this laboratory measurement without seeking either radiologic or histologic evidence may be overly sensitive and register many false-positive cases. Meanwhile, physicians noting that hyperamylasemia was a nonspecific phenomenon among uremic patients would not adequately treat these individuals.

In order to overcome this misclassification bias, we restricted our inclusion to those patients who had been hospitalized, as there should be less false positive cases. In studies using the same database, [11], [20] the positive predictive value was reasonably high (90.0%) among randomly selected hospitalized patients coded with AP. However, some AP patients may be cared for in the emergency departments (ED) until hospital beds become available (a process known as ED boarding). Patients who had mild AP may be discharged directly from the ED without hospitalization after several days if conditions improved. Our selection process decreased the generalizability of study findings, but we had decided this a mandatory trade-off. Similarly, we did not report the annual incidence of AP, because it would inevitably be lower than the true incidence.

Second, although the database included numerous comorbidities, it does not include presumed etiology of each AP attack. The composition of AP etiologies may be quite different between uremic patients and the general population. Although the lack of information did not bias results of this study, we recognize that knowledge of the composition leads to further understanding of the pathophysiology. Future individual-based cohort studies will be needed to address this limitation.

Third, alcohol abuse was assumed to be the cause of AP, especially in patients reporting >60 g pure alcohol/day. The study cohort precluded our ability to ascertain daily alcohol consumption; neither do we have information related to the prevalence of alcohol dependence for either study groups. The best effort we could have done was to approximate the alcohol use by the co-existing alcohol intoxication disease code at discharge. The prevalence of the alcohol intoxication code in the uremic group does not differ significantly from that seen in the control group. An “Alcohol diary” kept by patients would more precisely characterize this risk factor in future studies.

Finally, the AP attack severity was not addressed in this study. It is possible to use item reimbursement to estimate the need for intensive care unit admission, ventilator use, or procedure for cyst drainage. However, these issues are outside of the scope of this study.

## Conclusions

The risk of hospital admission due to AP is three times higher in patients receiving HD than in the general population over the four-year study period. In addition, the 7.3% mortality rate in the HD group is also higher than the 2.1% of the general population. Clinicians who treat HD patients who complain of abdominal pain should be aware of this possible diagnosis.
